# Personal and Family History of Cancer and Primary Lung Cancer Prevalence Among Never Smoking Disaggregated Asian American Women

**DOI:** 10.3390/cancers18121862

**Published:** 2026-06-06

**Authors:** Bani Kaur, Avinav Biswas, Tyler Chervo, Woo Jin Ahn, Shangzi Gao, Dang Nguyen, Carissa A. Villanueva, Seth J. Tivakaran, Malathi Srinivasan, Nicholas L. Panyanouvong, Lester Andrew V. Uy, Nitya Rajeshuni, Robert J. Huang, Neil Kamdar, Osamu Yasui, Gloria S. Kim, Latha Palaniappan, Jeffrey B. Velotta

**Affiliations:** 1 Stanford University School of Medicine, Palo Alto, CA 94305, USA; kaurbani@stanford.edu (B.K.); avinav@stanford.edu (A.B.); wjahn@stanford.edu (W.J.A.); szgao17@stanford.edu (S.G.); kevin_nguyen@hsph.harvard.edu (D.N.);; 2 Department of Medicine, Stanford Center for Asian Health Research and Education, Palo Alto, CA 94304, USA; 3 Division of Research, Kaiser Permanente Northern California, Pleasanton, CA 94588, USA; 4 Harvard T.H. Chan School of Public Health, Harvard University, Boston, MA 02115, USA; 5 Department of Surgery, University of California, San Francisco-East Bay, Oakland, CA 94602, USA; 6 Cecil G. Sheps Center for Health Services Research, University of North Carolina, Chapel Hill, NC 27599, USA; 7 Institute for Healthcare Policy and Innovation, University of Michigan, Ann Arbor, MI 48109, USA; 8 Department of Surgery, UCSF School of Medicine, San Francisco, CA 94143, USA; 9 Department of Clinical Sciences, Kaiser Permanente Bernard J. Tyson School of Medicine, Pasadena, CA 91101, USA; 10 Kaiser Permanente Northern California Oakland, Oakland, CA 94612, USA

**Keywords:** lung cancer, never-smokers, Asian American, health disparities, history of cancer

## Abstract

Background: Despite a decline in lung cancer in the U.S., lung cancer among never-smoking Asian American (AsA) women is rising, and subgroup aggregation obscures heterogeneity. We compared primary lung cancer prevalence across disaggregated AsA subgroups and examined factors associated with prevalence such as personal- and family-cancer histories versus Non-Hispanic Whites (NHWs). Methods: This cross-sectional study analyzed electronic health records of AsA women (≥18) in a large Northern California health system (2010–2022). Lung cancer cases were obtained from the hospital registry and categorized by smoking status and self-reported ethnicity. Adjusted prevalence ratios (aPRs) were estimated using targeted maximum likelihood estimation, accounting for sociodemographic, smoking, and clinical covariates. Results: Among 1,843,119 women, 8651 had primary lung cancer; 2429 were never-smokers. In AsA never-smokers, aPRs and 95% confidence intervals versus age-matched NHW were: Chinese (3.36, [3.20–3.53]), Filipino (2.68, [2.55–2.82]), Vietnamese (2.07, [1.96–2.18]), Japanese (1.99, [1.89–2.10]), Korean (1.90, [1.80–2.00]), and Other Asian (0.35, [0.33–0.37]). Personal cancer-history reflected an increase in prevalence among Korean patients (2.91, [2.76–3.06]) while family cancer-history demonstrated increased prevalence among Chinese patients (1.51, [1.42–1.60]). Among women with uterine cancer, Chinese patients had higher lung-cancer prevalence than NHW (1.91, [1.58–2.31]). Conclusions: Never-smoking disaggregated AsA women show heterogeneous lung cancer prevalence, with higher prevalence in Korean women with personal cancer-history and in Chinese women with family cancer-history compared with NHW, supporting history-informed and ethnic-specific lung cancer screenings.

## Simple Summary

The rise in the prevalence of primary lung cancer in never-smoking Asian American women compared to their Non-Hispanic White (NHW) counterparts suggests additional factors associated with prevalence (personal or family history of cancer) in this demographic. However, the aggregation of a large, diverse group of Asian Americans often obscures the heterogeneity and specific factors associated with prevalence in a certain ethnicity. In this study, we analyzed the association of having a family or personal history of cancer to explain the high prevalence of lung cancer in disaggregated Asian Americans (Chinese, Japanese, Korean, Vietnamese, Filipino, and Other Asian). We found that Korean women with a personal history of any cancer and Chinese women with a family history of any cancer have a higher prevalence of primary lung cancer compared to their NHW counterparts. Our findings call for history-informed and ethnic-specific lung cancer screenings to identify higher-prevalence subgroups.

## 1. Introduction

Lung cancer is the most frequently diagnosed cancer and the leading cause of cancer mortality worldwide, accounting for an estimated 2.5 million new cases and 1.8 million deaths in 2022, including approximately 908,630 new cases among women [1,2]. Adenocarcinoma was the predominant histologic subtype among both women and men in 2022, accounting for 59.7% of female lung cancer cases and 45.6% of male lung cancer cases [2]. If 2022 incidence and mortality rates remain stable, the global lung cancer burden is projected to reach 4.62 million new cases and 3.55 million deaths annually by 2050 [3]. Within this growing burden, never-smoking women represent an increasingly important population, particularly Asian American (AsA) women, among whom lung cancer mortality remains high across several ethnic subgroups [4]. While smoking is a well-established cause of lung cancer, a significant proportion of primary lung cancer occurred in never-smokers, with a California-based study reporting that 15% of primary lung cancer cases were in this population [5]. Notably, if never-smoking lung cancer was considered an independent category, it would rank among the top 10 causes of cancer death in the nation [6]. Additionally, as smoking rates continue to decrease, a study predicted that never-smoking lung cancer rates may eventually be more common than smoking lung cancer cases [7].

Multiple studies have reported that females appear disproportionately affected by never-smoking lung cancer, with an analysis reporting roughly twice the diagnosis rate observed in men [5]. Moreover, a California population-based study found incidence about twofold higher in Asian and Pacific Islander populations than in Non-Hispanic White (NHW), Black, or Hispanic populations, with a steeper temporal increase in the former group [8]. Despite this burden, U.S. screening guidelines rely on smoking-related metrics (pack-years and years since quitting), leaving most never-smokers ineligible [9] and resulting in more than 75% of lung cancers in AsAs going undetected under current United States Preventive Services Task Force criteria [10]. Among AsAs who are screening-eligible, recent KPNC data further demonstrate that screening uptake remains low and varies substantially across disaggregated AsA subgroups [11].

Most existing lung cancer screening tools were developed in cohorts of heavy smokers and therefore do not capture exposures that matter for never-smokers, including second-hand smoke, cooking-oil fumes, radon, and tuberculosis [12,13,14]. In addition, a growing body of work implicates a family history of lung cancer as a determinant of lung cancer prevalence. For instance, the TALENT study found an elevated risk of lung cancer for participants who have a family history of lung cancer, a risk that increases with the number of affected first-degree relatives, especially for mothers and siblings with lung cancer [15]. Consistent with these observations, a study reported that Asian and Pacific Islander women with lung cancer were three times more likely than never-smoking controls to have a documented family history of the disease [16], and a Korean population-based analysis using KoGES data found that a family history of lung cancer in first-degree relatives was associated with higher odds of lung cancer, with the association most evident among women, never-smokers, and younger individuals [17]. Additionally, prior breast, head, and neck cancer along with prior therapeutic radiation, chronic lung disease, and EGFR mutation-positive tumors have been associated with lung cancer prevalence in never-smokers; the diversity of exposures and pathways suggests a complex etiology that remains difficult to resolve [18,19,20,21,22]. This heterogeneity is summarized in a recent comprehensive review of the epidemiologic, genomic, and clinical landscape of lung cancer in never-smokers [23].

Given the heterogeneity of the Asian population, a Northern California study found that in a disaggregated AsA cohort, there was a higher incidence of primary lung cancer in all AsA ethnicities with the exception of those of Japanese descent [22]. However, these associations have not been systematically evaluated across disaggregated Asian subgroups with a family or personal history of cancer. In a companion analysis of the same integrated health system, Villanueva et al. recently reported that a personal or family history of cancer was associated with increased primary lung cancer prevalence among adult women overall, regardless of race, ethnicity, or smoking status [24]. The present study extends that work in three distinct directions: it is restricted to never-smoking women, it disaggregates the Asian American population into six self-reported ethnic subgroups, and it estimates ethnicity-specific adjusted prevalence ratios relative to Non-Hispanic White women rather than relative to women without a prior cancer history. We hypothesize that AsA women with a personal or family history of cancer have a greater prevalence of primary lung cancer than their NHW counterparts, and that this association varies meaningfully across AsA subgroups.

## 2. Methods

### 2.1. Data Source

Data for this analysis were drawn from the Kaiser Permanente Northern California (KPNC) integrated delivery system, which provides comprehensive care to a membership of over 4.5 million enrollees across the Northern and Central California regions and has been described in detail elsewhere [24,25]. As KPNC functions concurrently as insurer and provider, its electronic health record captures longitudinal in-network utilization across inpatient, emergency, outpatient, ambulatory, laboratory, and pharmacy encounters with high completeness. Incident cancer diagnoses are recorded in a SEER-compliant institutional registry that additionally collects tumor characteristics, treatment, and survival information [26]. This study was approved by the Kaiser Permanente Northern California Institutional Review Board (IRB) with a waiver of written informed consent. The IRB number is 2182779-4 and was approved on 24 February 2025.

### 2.2. Study Population

The primary purpose of this study was to estimate the adjusted prevalence ratio (aPR) of new primary lung cancer cases among AsA women relative to NHW women. We conducted a retrospective cross-sectional analysis covering 1 January 2010 through 31 December 2022. For each calendar year of the study period, a woman was considered eligible if, as of 1 January of that year (her index date), she was at least 18 years of age, self-identified as AsA or NHW, had no prior diagnosis of primary lung cancer, and had accrued at least nine months of KPNC membership in the preceding 12 months. Women who first met all criteria in more than one year were entered into the analytic cohort using the earliest qualifying 1 January as their index date. Kaiser Permanente maintains a SEER compliant cancer registry that was used to identify the outcome of lung cancer cases for this study.

### 2.3. Exposures

The primary exposure was self-reported AsA ethnicity, obtained from self-reported data in patient charts. The specific AsA ethnicities that had a sufficient sample size to study were Chinese, Japanese, Filipino, Korean, and Vietnamese, each compared with NHW women as the reference group. All other AsA ethnicities, including Asian Pacific Islanders, Hmong, Afghan, Burmese, Cambodian, Fijian, Nepalese, Pakistani, Southeast Asian, South Asian, Indonesian, Laotian, East Asian, Thai, Taiwanese, and those of multiple Asian ethnicities, were grouped into “Other Asian.”

Secondary exposures were a personal history of any cancer, breast cancer, cervical cancer, uterine cancer, or ovarian cancer, and a family history of any cancer, breast cancer, or lung cancer. A personal history of the following gynecological cancers common in women was ascertained using a combination of International Classification of Disease (ICD) version 9 and 10 codes and the KPNC cancer registry for cervical, uterine, breast, and ovarian cancer. Given the association of a family history of lung cancer with increased primary lung cancer prevalence reported in the TALENT study [15], a family history of lung cancer and breast cancer was also assessed using ICD-9 and ICD-10 codes. A personal history of any type of cancer was assessed using a combination of ICD-9 and ICD-10 codes together with any entry in the KPNC cancer registry. A family history of any cancer was assessed using ICD-9 and ICD-10 codes. A complete list of ICD-9 and ICD-10 codes used in this study is provided in Appendix A.

### 2.4. Outcome

The outcome for all analyses was incident primary lung cancer occurring on or after the index date, identified from the KPNC SEER-compliant cancer registry using ICD-O-3 site codes C340 through C349.

### 2.5. Covariates

We additionally ascertained the following covariates: Age in years at first date of eligibility for the study, race/ethnicity, smoking history, Charlson Comorbidity Index category, body mass index (BMI) category, and a history of type II diabetes (Yes/No). The Charlson Comorbidity Index was implemented as a weighted combination of conditions documented in the one-year look-back period preceding each patient’s first year of eligibility, following the implementation described by Charlson et al. [27]. The ‘No visits in the past year’ category refers to patients who had no KPNC encounters during this one-year look-back window and therefore had no opportunity to accrue diagnosis codes. Smoking status was captured from a standardized questionnaire administered during routine KPNC outpatient encounters, and all remaining covariates were derived from the electronic health record. For women with the outcome, personal and family history of cancer were ascertained from records dated before the lung cancer diagnosis; for women without the outcome, they were ascertained from records dated at any point within the study window. Where multiple values were available, the measurement closest in time to the index date was used.

### 2.6. Statistical Analysis

Adjusted prevalence ratios were estimated using Targeted Maximum Likelihood Estimation (TMLE), a doubly robust semiparametric framework that targets a prespecified marginal parameter, here the covariate-adjusted prevalence ratio, while permitting flexible nuisance estimation [28]. Nuisance functions for the outcome regression and propensity score were fit using a SuperLearner ensemble in which candidate algorithms are combined through cross-validated weights to minimize prediction error [29]. The candidate library comprised generalized linear models, LASSO-penalized regression, extreme gradient boosting (xgboost), and an intercept-only mean learner serving as a negative control. A comparative description of TMLE relative to conventional logistic regression is provided in Appendix C.

All TMLE models were adjusted for age, race/ethnicity, Charlson Comorbidity Index category, body mass index category, and history of type II diabetes. Each model compared one AsA subgroup with NHW women (the reference group) within a single, prespecified stratum. Strata were defined by (i) smoking status (never, current, or former), (ii) any personal history of cancer, any family history of cancer, or the combination of both, and (iii) site-specific personal history (breast, uterine, cervical, or ovarian) and site-specific family history (breast or lung) of cancer. In models stratified by a site-specific personal or family history of cancer (e.g., breast cancer), we additionally adjusted for the remaining site-specific cancer histories considered in this study. Across all strata, the analytic plan comprised 66 pairwise AsA-versus-NHW comparisons.

We utilized the Bonferroni correction to control the family-wise error rate for multiple comparisons by setting the *p*-value threshold for statistical significance at 0.05 divided by the 66 comparisons conducted, yielding α ≈ 7.6 × 10^−4^. Data extraction was performed using SAS Version 9.4 (SAS Institute, Cary, NC, USA) and the analysis was done using the R programming language version 4.3.1 (R Foundation for Statistical Computing, Vienna, Austria) with the tidyverse and tmle3 packages.

## 3. Results

### 3.1. Cohort Assembly and Baseline Characteristics

**Figure 1:**
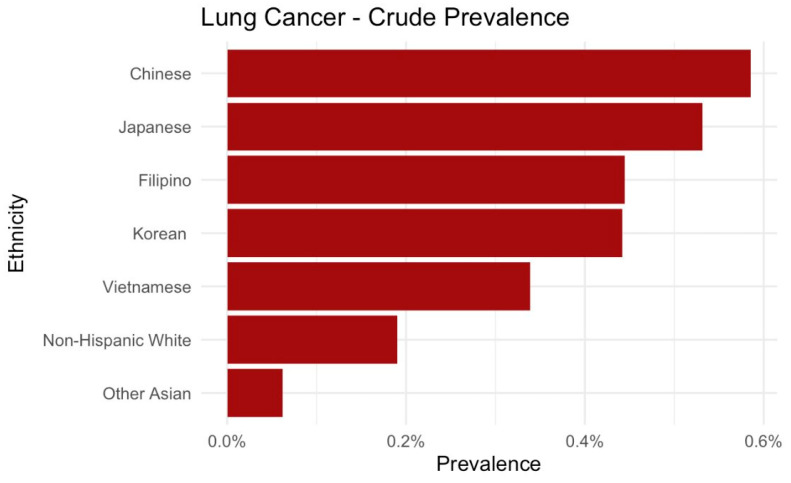
Crude prevalence of never-smoking lung cancer. Crude prevalence was calculated as the number of never-smoking women with primary lung cancer divided by the total number of eligible never-smoking women in the same race/ethnicity subgroup, expressed as a percentage. For example, among Chinese women, crude prevalence was 379/64,695 = 0.586%.

From 2010 to 2022, 1,843,119 women met the inclusion criteria for this study. Linkage to the institutional cancer registry identified 8651 women with primary lung cancer; 2429 were never-smokers. In AsA women, we identified 961 never smoking cases with 1354 total primary lung cancer cases. The analytic cohort was stratified by smoking status and self-reported AsA ethnicity (Chinese, Japanese, Filipino, Korean, Vietnamese, Other Asian), with NHW women as the reference group. Distributions of age, body mass index, Charlson Comorbidity Index, type II diabetes, and smoking status for the full cohort and for women with prevalent lung cancer along with ethnic subgroup counts and the proportion of never/former/current smokers are provided in Table 1, with crude prevalence by ethnicity shown in Figure 1, and unadjusted counts and prevalence by personal and family cancer history summarized in Table 2.

### 3.2. Ethnicity-Specific Prevalence in Never-Smokers

**Figure 2:**
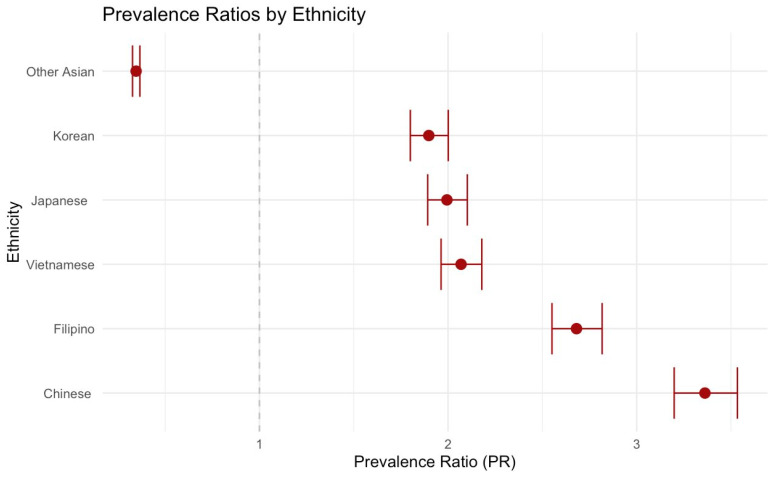
Prevalence ratios for primary lung cancer among never-smoking AsA women compared with never-smoking NHW women. Points represent adjusted prevalence ratios, and horizontal bars represent 95% confidence intervals. The dashed vertical line indicates the NHW reference value of 1.0. Each estimate compares eligible never-smoking women in the specified AsA subgroup with eligible never-smoking NHW women.

Among never-smokers, there was substantial heterogeneity in aPRs relative to age-matched NHW women serving as our reference. The aPR was highest in Chinese women (3.36, 95% CI 3.20–3.53), followed by Filipino (2.68, 95% CI 2.55–2.82), Vietnamese (2.07, 95% CI 1.96–2.18), Japanese (1.99, 95% CI 1.89–2.10), and Korean (1.90, 95% CI 1.80–2.00). In contrast, women classified as Other Asian had a lower adjusted prevalence (0.35, 95% CI 0.33–0.37). These estimates are displayed in a forest plot in Figure 2 and detailed in Table 3.

### 3.3. Personal History of Cancer

When stratifying by personal history of any cancer, Korean women demonstrated a markedly higher adjusted prevalence of lung cancer compared with their NHW counterparts (aPR 2.91, 95% CI 2.76–3.06), as shown in Table A1 (Appendix B). In analyses focused on specific prior cancers, a personal history of uterine cancer was associated with a higher prevalence among Chinese women (aPR 1.91, 95% CI 1.58–2.31), while analogous elevations were not observed with consistency across other ethnic subgroups. Full estimates across personal history strata are reported in Table A2 (Appendix B).

### 3.4. Family History of Cancer

A family history of any cancer was associated with higher adjusted prevalence among Chinese women (aPR 1.51, 95% CI 1.42–1.60). Patterns for other ethnicities were less uniform and are presented in Table A3 (Appendix B).

## 4. Discussion

Building on a recent companion analysis from the same integrated health system that established a positive association between any personal or family cancer history and lung cancer prevalence in adult women overall [24], the present study is the first to evaluate this association across disaggregated AsA subgroups of never-smoking women and to benchmark each subgroup against Non-Hispanic White women within the same cancer-history stratum. In addition, this study is novel in that we present detailed machine learning models to ascertain lung cancer prevalence for each disaggregated Asian American subgroup after adjusting for clinical and sociodemographic variables and believe this work adds to the growing notion that each Asian American subgroup is unique. Consistent with the findings in De Rouen et al. 2022 and Banks et al. 2022, our results demonstrated that never-smoking female AsA subgroups had a higher prevalence of primary lung cancer compared to their NHW counterparts [5,22]. Additionally, out of the total lung cancer cases stratified by ethnicity, there is a greater proportion of never-smokers in AsA populations, with the lowest seen in NHW and other Asians. This finding could be explained by Banks et al. 2022 which found that AsAs were less likely to smoke compared to their NHW counterparts [5]. However, unlike other studies, our disaggregated prevalence ratios revealed that Chinese women display the highest prevalence compared to their AsA counterparts. In this context, our results underscore the need to reevaluate lung cancer screening guidelines to recognize ethnic-specific prevalence patterns in never-smoking populations, particularly within heterogeneous AsA groups.

Evaluating the association of a personal or family history of a previous type of cancer with this elevated prevalence in AsAs revealed that having a personal history of any type of cancer was associated with a greater prevalence ratio of primary lung cancer in Koreans compared to their NHW counterparts. However, Chinese, Filipino, Vietnamese, and Other Asians had a lower prevalence ratio, indicating that while having a personal history of any type of cancer is associated with higher prevalence in Korean women, this does not explain the greater prevalence observed in other AsA ethnicities, suggesting that each ethnicity demonstrates an increased prevalence attributable to various reasons. The unexpectedly low adjusted prevalence ratios observed for several subgroups with a personal history of cancer, despite the crude prevalence values shown in Table 2, likely reflect sparse outcome counts within ethnicity-by-cancer-history strata rather than reflecting a true protective association. These estimates should therefore be interpreted cautiously. Similar to Nofal et al. 2024 who analyzed the association of personal histories of cancer with primary lung cancer, we disaggregated by type of cancer to analyze if certain cancers explain the increased prevalence in AsA [19]. Focusing on uterine cancer for its statistical and clinical significance and sufficient sample size, we found that prior uterine cancer was associated with a greater prevalence of primary lung cancer in Chinese women compared to their NHW counterparts, indicating that uterine cancer is associated with a higher prevalence of primary lung cancer in Chinese women, but does not apply for other ethnicities. While Wang et al. 2021 uncovered a personal history of breast cancer to be a contributing factor to primary lung cancer prevalence, we were unable to determine this association in our study cohort due to the limited sample size [18]. Recent whole-genome and whole-exome sequencing from the Women’s Health Initiative has further shown that lung adenocarcinoma in postmenopausal never-smoking women has mutational patterns that diverge from those observed in smokers, supporting the presence of distinct etiologic pathways in this population [30]. Similar to the analysis for Korean women, we were unable to determine what types of cancers explain the greater prevalence of primary lung cancer but our results suggest Koreans and Chinese with a personal history of cancer and a personal history of uterine cancer, respectively, are higher-prevalence subgroups. Similar to Nofal et al. 2024, we suggest that personal history of cancer be recognized as an eligibility criterion for primary lung cancer screening [19]. Given the National Lung Screening Trial’s success with low-dose computed tomography (CT) in identifying and reducing lung cancer-associated deaths in high-prevalence populations, personal and family cancer history coupled with ethnic-based history should be recognized as a formal factor in screening eligibility [31].

We further analyzed the association of a family history of cancer with the high prevalence of primary lung cancer in AsAs. We found that Chinese females with a family history of any type of cancer had a higher prevalence of primary lung cancer compared to their NHW and AsA counterparts, suggesting differentiated prevalence patterns. Given that the TALENT trial found that a family history of lung cancer was associated with increased primary lung cancer prevalence, we further stratified by type of family history of cancer [15]. Our sample size limited our ability to stratify by specific cancer types; thus, we could not determine if the observed elevated prevalence in Chinese women with a family history of cancer was driven by specific cancers. Larger, multi-site studies will be necessary to disentangle these relationships.

## 5. Limitations

We acknowledge three limitations. First, although our study population is drawn from Northern California, a region with one of the most diverse Asian populations globally, the findings may not be generalizable nationwide. Nevertheless, the KPNC lung cancer database has been recognized as one of the most representative in the U.S. in a study by Yang et al., 2024, supporting the relevance of our cohort to broader national trends in lung cancer epidemiology [25]. Subsequently, due to the retrospective nature of our analysis, there is an inherent risk of missing or incomplete clinical data which may obscure our findings.

Second, while we examined the association of a personal and family history of cancer with lung cancer prevalence, environmental exposures such as second-hand smoke, radiation, and cooking fumes are associated with lung cancer and are difficult to quantify. To address this, we applied TMLE to estimate aPRs that accounted for confounding variables, providing a clearer prevalence estimate.

Last, the limited sample size required the aggregation of several AsA ethnicities into a single cohort, underscoring the need for future studies focused on the diversity of minority AsA populations. Additionally, specific types of family cancer histories could not be analyzed separately, preventing us from determining whether specific cancer types drive the elevated prevalence observed in certain AsA subgroups. Larger cohorts are needed to clarify these relationships. Additionally, the data extraction for this analysis covered 2010 through 2022; subsequent calendar years were not available with sufficient cancer registry completeness at the time of analysis and will be incorporated in future work.

## 6. Conclusions

Our study is the first to evaluate how a personal and family history of cancer influences primary lung cancer prevalence in disaggregated AsA women. Across never-smoking women in an integrated U.S. system, AsA subgroups showed higher prevalence than NHW women, with the highest prevalence in Chinese women. A personal history of any cancer identified Korean women at an elevated prevalence, while family history identified Chinese women. The association with uterine cancer history in Chinese women highlights etiologic heterogeneity. As this analysis is cross-sectional and prevalence-based, it cannot quantify incidence, absolute lung cancer risk, or the mortality benefit of screening, and these findings should therefore be interpreted as hypothesis-generating. Before disaggregated ethnicity and prior cancer history can be incorporated into formal lung cancer screening guidelines, prospective validation will be required, including the longitudinal estimation of absolute and relative incidence in independent cohorts, integration into externally validated risk-prediction models, and a dedicated assessment of low-dose CT screening yield, benefits, harms, and cost-effectiveness within each subgroup.

## Figures and Tables

**Table 1:** Demographic, Clinical, and Smoking Characteristics of the KPNC Cohort and of Women with Prevalent Primary Lung Cancer, by Smoking Status.

	Cohort				Cohort Prevalent Lung Cancer Cases			
Characteristic	Never, N = 1,184,772 ^1^	Former, N = 217,830 ^1^	Current, N = 123,079 ^1^	Unknown, N = 317,438 ^1^	Never, N = 2429 ^1^	Former, N = 3139 ^1^	Current, N = 2160 ^1^	Unknown, N = 923 ^1^
Demographic characteristics								
Age (yrs) at anchor date (standard deviation)	42 (18)	53 (17)	43 (16)	40 (18)	64 (13)	70 (9)	64 (9)	65 (11)
Race/ethnicity								
NHW	748,405 (63%)	191,845 (88%)	104,032 (85%)	205,095 (65%)	1431 (59%)	2995 (95%)	2074 (96%)	749 (81%)
Asian	416,744 (35%)	23,468 (11%)	17,249 (14%)	106,753 (34%)	961 (40%)	139 (4.4%)	83 (3.8%)	171 (19%)
Analysis ethnicity								
White	746,921 (63%)	191,613 (88%)	103,885 (84%)	204,640 (64%)	1420 (58%)	2994 (95%)	2071 (96%)	749 (81%)
Chinese	64,695 (5.5%)	2664 (1.2%)	1553 (1.3%)	16,224 (5.1%)	379 (16%)	30 (1.0%)	12 (0.6%)	62 (6.7%)
Filipino	71,292 (6.0%)	7682 (3.5%)	5347 (4.3%)	17,963 (5.7%)	317 (13%)	45 (1.4%)	26 (1.2%)	46 (5.0%)
Japanese	8842 (0.7%)	2029 (0.9%)	684 (0.6%)	2526 (0.8%)	47 (1.9%)	32 (1.0%)	20 (0.9%)	16 (1.7%)
Korean	7246 (0.6%)	807 (0.4%)	575 (0.5%)	1963 (0.6%)	32 (1.3%)	7 (0.2%)	11 (0.5%)	4 (0.4%)
Other Asian	265,119 (22%)	12,491 (5.7%)	10,548 (8.6%)	69,490 (22%)	164 (6.8%)	29 (0.9%)	18 (0.8%)	34 (3.7%)
Vietnamese	20,657 (1.7%)	544 (0.2%)	487 (0.4%)	4632 (1.5%)	70 (2.9%)	2 (<0.1%)	2 (<0.1%)	12 (1.3%)
BMI								
Normal weight	543,824 (46%)	74,734 (34%)	45,358 (37%)	90,476 (29%)	1105 (45%)	1077 (34%)	882 (41%)	259 (28%)
Underweight	72,538 (6.1%)	4675 (2.1%)	4951 (4.0%)	17,572 (5.5%)	79 (3.3%)	100 (3.2%)	154 (7.1%)	27 (2.9%)
Overweight	271,983 (23%)	58,328 (27%)	30,810 (25%)	45,925 (14%)	673 (28%)	939 (30%)	562 (26%)	177 (19%)
Obese	253,503 (21%)	74,679 (34%)	37,295 (30%)	42,902 (14%)	537 (22%)	985 (31%)	511 (24%)	184 (20%)
Unknown	42,924 (3.6%)	5414 (2.5%)	4665 (3.8%)	120,563 (38%)	35 (1.4%)	38 (1.2%)	51 (2.4%)	276 (30%)

^1^ Descriptive statistics of patient demographic, clinical characteristics, and smoking status were computed using frequency and percentage for categorical variables and mean and standard deviation for continuous variables.

**Table 2:** Unadjusted Counts and Prevalence of Primary Lung Cancer, by Ethnicity and Personal or Family History of Any Cancer.

Strata	N Total	N with Outcome	Unadjusted Prevalence
Personal history of any cancer			
White	109,576	1431	0.0131
Chinese	6222	60	0.0096
Filipino	9115	70	0.0077
Japanese	1722	23	0.0134
Korean	875	10	0.0114
Vietnamese	1371	10	0.0073
Other Asian	8392	29	0.0035
Family history of any cancer			
White	173,439	1003	0.0058
Chinese	9525	57	0.0060
Filipino	11,721	39	0.0033
Japanese	2071	12	0.0058
Korean	1172	3	0.0026
Vietnamese	2383	4	0.0017
Other Asian	22,426	28	0.0012
Personal and family history of any cancer			
White	27,838	286	0.0103
Chinese	1369	14	0.0102
Filipino	2042	7	0.0034
Japanese	435	3	0.0069
Korean	162	1	0.0062
Vietnamese	265	0	0.0000
Other Asian	1402	6	0.0043

**Table 3:** Adjusted Prevalence Ratios for Primary Lung Cancer Among Never-Smoking Asian American Women, by Ethnicity (Reference: Non-Hispanic White Women).

Ethnicity	Prevalence Ratio (95% CI)	*p*-Value
Chinese	3.362 (3.199, 3.534)	<0.001
Japanese	1.994 (1.892, 2.102)	<0.001
Filipino	2.681 (2.551, 2.817)	<0.001
Korean	1.898 (1.8, 2.001)	<0.001
Vietnamese	2.069 (1.963, 2.179)	<0.001
Other Asian	0.346 (0.327, 0.366)	<0.001

All ethnicities are compared to NHW.

## Data Availability

The data obtained for this study was obtained from the Kaiser Permanente Northern California Healthcare system and the SEER cancer registry.

## Appendix A. International Classification of Disease (ICD) Codes Used to Identify Personal and Family History of Cancers

**History Type**	**ICD Version**	**Codes**
Family history of any cancer	ICD-9	V16.0, V16.1 V16.2, V16.3 V16.4, V16.5, V16.6, V16.7, V16.8, V16.9
	ICD-10	Z80.0, Z80.1 Z80.2, Z80.3, Z80.4, Z80.5, Z80.6, Z80.7, Z80.8, Z80.9
Family history of breast cancer	ICD-9	V16.3
	ICD-10	Z80.3
Family history of lung cancer	ICD-9	V16.1
	ICD-10	Z80.1
Personal history of any cancer	ICD-9	V10.0, V10.1, V10.2, V10.3, V10.4, V10.5, V10.6, V10.7, V10.8, V10.9
	ICD-10	Z85
History of breast cancer	ICD-9	V10.3
	ICD-10	Z85.3
History of cervical cancer	ICD-9	V10.41
	ICD-10	Z85.1
History of ovarian cancer	ICD-9	V10.43
	ICD-10	Z85.43
History of uterine cancer	ICD-9	V10.42
	ICD-10	Z85.42
Outcome Lung Cancer	ICD-O-3	C340–C349
The ICD-9, ICD-10, and ICD-O-3 code definitions used here were developed for and previously published by Villanueva et al. [24] and are reproduced with the authors’ permission to ensure consistency in case ascertainment across the two companion analyses.		

## Appendix B. Adjusted Prevalence Ratios Stratified by Personal and Family History of Cancer

**Table A1:** Prevalence Ratios for Primary Lung Cancer by Ethnicity and Any Personal or Family History of Cancer.

Ethnicity	Cancer Type	Prevalence Ratio (95% CI)	*p*-Value
Chinese	PH of any cancer	0.153 (0.148, 0.157)	<0.001
Chinese	FH of any cancer	1.507 (1.418, 1.602)	<0.001
Japanese	PH of any cancer	1.001 (0.953, 1.052)	0.953
Japanese	FH of any cancer	0.951 (0.893, 1.012)	0.114
Filipino	PH of any cancer	0.073 (0.071, 0.076)	<0.001
Filipino	FH of any cancer	0.769 (0.723, 0.818)	<0.001
Korean	PH of any cancer	2.909 (2.764, 3.061)	<0.001
Korean	FH of any cancer	0.598 (0.561, 0.636)	<0.001
Vietnamese	PH of any cancer	0.222 (0.213, 0.23)	<0.001
Vietnamese	FH of any cancer	0.489 (0.459, 0.521)	<0.001
Other Asian	PH of any cancer	0.042 (0.04, 0.044)	<0.001
Other Asian	FH of any cancer	0.315 (0.295, 0.337)	<0.001

PH = personal history; FH = family history. All ethnicities are compared to NHW. Cancer type represents the stratification for these patients that have either a personal or family history of any cancer. For example, in the first row of Table A1, the aPR is the comparison among Chinese patients with a personal history of any cancer and NHW with a personal history of any cancer.

**Table A2:** Prevalence Ratios for Primary Lung Cancer, by Ethnicity and Personal History of Site-Specific Cancer.

Ethnicity	Cancer Type	Prevalence Ratio (95% CI)	*p*-Value	Bonferroni Adjusted Significance
Chinese	PH of breast cancer	0.132 (0.126, 0.137)	*p* < 0.001	TRUE
Chinese	PH of uterine cancer	1.908 (1.575, 2.31)	*p* < 0.001	TRUE
Chinese	PH of cervical cancer	0.55 (0.451, 0.671)	*p* < 0.001	TRUE
Chinese	PH of ovarian cancer	0.932 (0.736, 1.179)	0.557	FALSE
Japanese	PH of breast cancer	1.167 (1.096, 1.244)	*p* < 0.001	TRUE
Japanese	PH of uterine cancer	0.82 (0.673, 0.999)	0.049	FALSE
Japanese	PH of cervical cancer	0 (0, 0)	*p* < 0.001	TRUE
Japanese	PH of ovarian cancer	0 (0, 0)	*p* < 0.001	TRUE
Filipino	PH of breast cancer	0.082 (0.079, 0.086)	*p* < 0.001	TRUE
Filipino	PH of uterine cancer	1.066 (0.88, 1.29)	0.515	FALSE
Filipino	PH of cervical cancer	0.177 (0.154, 0.205)	*p* < 0.001	TRUE
Filipino	PH of ovarian cancer	0.492 (0.404, 0.599)	*p* < 0.001	TRUE
Korean	PH of breast cancer	1.476 (1.383, 1.576)	*p* < 0.001	TRUE
Korean	PH of uterine cancer	0 (0, 0)	*p* < 0.001	TRUE
Korean	PH of cervical cancer	0.977 (0.781, 1.223)	0.841	FALSE
Korean	PH of ovarian cancer	2.862 (2.09, 3.918)	*p* < 0.001	TRUE
Vietnamese	PH of breast cancer	0.28 (0.266, 0.295)	*p* < 0.001	TRUE
Vietnamese	PH of uterine cancer	0 (0, 0)	*p* < 0.001	TRUE
Vietnamese	PH of cervical cancer	0.833 (0.669, 1.037)	0.102	FALSE
Vietnamese	PH of ovarian cancer	0 (0, 0)	*p* < 0.001	TRUE
Other Asian	PH of breast cancer	0.056 (0.053, 0.058)	*p* < 0.001	TRUE
Other Asian	PH of uterine cancer	0.174 (0.14, 0.216)	*p* < 0.001	TRUE
Other Asian	PH of cervical cancer	2.561 (2.203, 2.977)	*p* < 0.001	TRUE
Other Asian	PH of ovarian cancer	1.895 (1.64, 2.189)	*p* < 0.001	TRUE

Statistical significance was defined using a Bonferroni-corrected threshold of α = 0.05/66 ≈ 7.6 × 10^−4^ to control the family-wise error rate across all pairwise comparisons in this study. TRUE indicates the result meets this threshold; FALSE indicates it does not.

**Table A3:** Prevalence Ratios for Primary Lung Cancer, by Ethnicity and Family History of Site-Specific Cancer.

Ethnicity	Cancer Type	Prevalence Ratio (95% CI)	*p*-Value	Bonferroni Adjusted Significance
Chinese	FH of breast cancer	1.114 (1.016, 1.222)	0.022	FALSE
Chinese	FH of lung cancer	0.245 (0.222, 0.27)	*p* < 0.001	TRUE
Japanese	FH of breast cancer	1.108 (1.009, 1.218)	0.032	FALSE
Japanese	FH of lung cancer	0.545 (0.468, 0.634)	*p* < 0.001	TRUE
Filipino	FH of breast cancer	0.605 (0.551, 0.665)	*p* < 0.001	TRUE
Filipino	FH of lung cancer	0.233 (0.206, 0.263)	*p* < 0.001	TRUE
Korean	FH of breast cancer	0 (0, 0)	*p* < 0.001	TRUE
Korean	FH of lung cancer	0 (0, 0)	*p* < 0.001	TRUE
Vietnamese	FH of breast cancer	0.316 (0.287, 0.347)	*p* < 0.001	TRUE
Vietnamese	FH of lung cancer	1.023 (0.875, 1.196)	0.773	FALSE
Other Asian	FH of breast cancer	0.342 (0.311, 0.378)	*p* < 0.001	TRUE
Other Asian	FH of lung cancer	0.077 (0.068, 0.088)	*p* < 0.001	TRUE

Statistical significance was defined using a Bonferroni-corrected threshold of α = 0.05/66 ≈ 7.6 × 10^−4^ to control the family-wise error rate across all pairwise comparisons in this study. TRUE indicates the result meets this threshold; FALSE indicates it does not.

## Appendix C. Targeted Maximum Likelihood Estimation: Comparison with Conventional Logistic Regression

Conventional multivariable logistic regression returns a conditional odds ratio that is non-collapsible and, in cohorts where the outcome is not rare, does not approximate a population-level prevalence ratio. It is also unbiased only when the parametric model is correctly specified. TMLE differs in three respects relevant to the present analysis. First, it targets a marginal parameter, the adjusted prevalence ratio, directly. Second, it is doubly robust: the estimator remains consistent if either the outcome regression or the propensity model is correctly specified. Third, it accommodates a data-adaptive estimation of these nuisance functions through the SuperLearner ensemble without inflating type I error, because a targeting step orthogonalizes the estimator with respect to first-order error in the nuisance estimates. These properties make TMLE preferable to logistic regression when (i) the population-level prevalence ratio rather than a conditional odds ratio is the parameter of interest, and (ii) the functional form linking covariates to outcomes is unknown.
